# Statistical Models for Predicting Threat Detection From Human Behavior

**DOI:** 10.3389/fpsyg.2018.00466

**Published:** 2018-04-16

**Authors:** Timothy Kelley, Mary J. Amon, Bennett I. Bertenthal

**Affiliations:** ^1^Naval Surface Warfare Center, Crane Division, Crane, IN, United States; ^2^Department of Psychological and Brain Sciences, Indiana University Bloomington, Bloomington, IN, United States

**Keywords:** threat detection, statistical models, phishing, mouse tracking, human dynamics, cyber security, cyberpsychology

## Abstract

Users must regularly distinguish between secure and insecure cyber platforms in order to preserve their privacy and safety. Mouse tracking is an accessible, high-resolution measure that can be leveraged to understand the dynamics of perception, categorization, and decision-making in threat detection. Researchers have begun to utilize measures like mouse tracking in cyber security research, including in the study of risky online behavior. However, it remains an empirical question to what extent real-time information about user behavior is predictive of user outcomes and demonstrates added value compared to traditional self-report questionnaires. Participants navigated through six simulated websites, which resembled either secure “non-spoof” or insecure “spoof” versions of popular websites. Websites also varied in terms of authentication level (i.e., extended validation, standard validation, or partial encryption). Spoof websites had modified Uniform Resource Locator (URL) and authentication level. Participants chose to “login” to or “back” out of each website based on perceived website security. Mouse tracking information was recorded throughout the task, along with task performance. After completing the website identification task, participants completed a questionnaire assessing their security knowledge and degree of familiarity with the websites simulated during the experiment. Despite being primed to the possibility of website phishing attacks, participants generally showed a bias for logging in to websites versus backing out of potentially dangerous sites. Along these lines, participant ability to identify spoof websites was around the level of chance. Hierarchical Bayesian logistic models were used to compare the accuracy of two-factor (i.e., website security and encryption level), survey-based (i.e., security knowledge and website familiarity), and real-time measures (i.e., mouse tracking) in predicting risky online behavior during phishing attacks. Participant accuracy in identifying spoof and non-spoof websites was best captured using a model that included real-time indicators of decision-making behavior, as compared to two-factor and survey-based models. Findings validate three widely applicable measures of user behavior derived from mouse tracking recordings, which can be utilized in cyber security and user intervention research. Survey data alone are not as strong at predicting risky Internet behavior as models that incorporate real-time measures of user behavior, such as mouse tracking.

## Introduction

Phishing is an attempt to steal an individual’s sensitive and personal information via social engineering and technical deception, such as mimicking a legitimate and trustworthy entity like a bank. These attacks often originate from emails—also designed to appear to come from a legitimate source—that contain links to malicious websites. Search engine results can also lead people to phishing websites (Myers, 2006; Ramzan, 2010). Phishing remains a significant risk despite many technical attempts to alert users to potential dangers. A recent report on the cost of phishing estimated the annual cost for a large company to be $3.7 million dollars (Ponemon Institute, 2015). Further investigation shows that there is a large market for personal data, with fresh credit card data fetching between 20 and 45 dollars per card (Ablon et al., 2014). The total number of phishing attacks in 2016 was 1,220,523 representing a 65% increase over 2015 (Anti-Phishing Working Group [APWG], 2016).

Email servers are designed to filter potential scam emails in order to protect users from phishing attempts. The filters are not perfect, however, and even though only a small portion of phishing emails reach individuals’ in-boxes, there are still enough individuals that click on the malicious links to sustain phishing fraud as a profitable venture (Kanich et al., 2008; Herley and Flor, 2010). While general phishing attacks have low success rates, attacks that utilize contextual information so it appears to come from a legitimate and known source greatly increases the likelihood the email will get through a filter and an individual will visit a malicious website and enter their information (Dhamija et al., 2006; Jagatic et al., 2007).

Web browsers use domain name and security indicators that identify whether users are at the intended website, whether a page is encrypted (i.e., electronic data is converted into a form that is not easily intercepted), and third party vetting of domain ownership (e.g., a website has extended validation). These indicators fail for a variety of reasons. Even web designers from popular websites often construct legitimate websites that behave in a manner similar to malicious websites. For example, some websites will redirect users to login screens that have a different domain name than the originating webpage, making it potentially difficult to for users identify the validity of the login screen (Stebila, 2010). If an individual has a history of visiting a given site, they will ignore warnings that the site is insecure, demonstrating more trust in the site than the browser’s alerts (Almuhimedi et al., 2014). Furthermore, the nature of information that security indicators communicate is somewhat technical and designed primarily for experts (Garg and Camp, 2012). These factors lead to increased uncertainty and many people, including technically savvy individuals, fall for phishing emails and enter their credentials at malicious websites on a daily basis (Jagatic et al., 2007; Stebila, 2010).

Despite the availability of security indicators, users are not guaranteed to understand, attend to, or respond appropriately to these cues. For example, Sheng et al. (2010) instructed participants to conduct banking tasks online, either role playing or using their real online credentials. Participants received increasingly alarming cues throughout the task, suggesting their connection was insecure. Cues ranged from a missing “https,” a missing site-authentication image, and a warning page. Participants who role-played the task were more likely to engage in risky online behavior, as compared to those who used their own accounts and passwords. However, even those using their real login information (92%) tended to ignore missing “https” and site-authentication images, suggesting that many security indicators are not effectively utilized (Schechter et al., 2007). In another study, participants navigating a series of simulated websites that varied in terms of security warnings successfully detected only 53% of phishing websites, even though they were explicitly instructed to identify the insecure websites (Alsharnouby et al., 2015). Moreover, Whalen and Inkpen (2005) found that participants displayed virtually no attention to security indicators without first priming them to the risk of malicious websites.

There have been numerous studies that examine technical and practical security knowledge as well as other demographic information that modulate the effectiveness of “spoof” websites, or hoax websites that appear to look like those belonging to a different person or organization. Many of these studies examine the relationship between an individual’s self-reported online activity, demographic characteristics, and knowledge of security through survey measures (Downs et al., 2007; Sheng et al., 2010; Wright and Marett, 2010). However, survey data reflecting, for example, self-reported security knowledge, is not necessarily a good predictor of behavior during phishing attacks. Kelley and Bertenthal (2016b) found that participants with high security knowledge were better than those with low security knowledge at identifying spoof websites, but were not better at identifying sites lacking encryption information. Furthermore, participants were more likely to login to non-spoof websites if they were familiar, but familiarity did not affect responses to spoof websites. These findings demonstrate that a high degree of security knowledge cannot ensure that users will consistently attend to and appropriately respond to all relevant factors that indicate security risk. Moreover, recent findings suggest that additional dynamically changing factors such as fatigue, cognitive load, and attention modulate the effects of security knowledge (e.g., Vishwanath et al., 2011, 2016; Kelley and Bertenthal, 2016a).

Researchers are increasingly looking to behavioral measures to gain a better understanding of users’ decision-making. Real-time measures such as eye movements, mouse movements, and physiological recordings like heart rate or pupillary responses can be used to quantify different aspects of decision-making. For example, Sobey et al. (2008) were among the first to employ eye-tracking technology to better understand the utilization of security indicators, finding that extended validation security indicators drew little attention from users without a modified web browser. By contrast, the ability to detect phishing websites is associated with an increase in gaze time directed toward these security indicators, such as a padlock or https (Alsharnouby et al., 2015); also, users with greater security expertise tend to gaze at security indicators longer (Arianezhad et al., 2013).

Mouse tracking is another viable measure of user behavior in that it does not typically require equipment outside of the computer interface and can be applied remotely (e.g., via Amazon’s Mechanical Turk data collection tool) (Dale and Duran, 2011). Rather than motor movements reflecting the final stage in decision making, motor movements are continuously updated based on cognitive processing, reflecting a thought process that evolves throughout a given task (Goodale et al., 1986; Cisek and Kalaska, 2005; Song and Nakayama, 2006; Freeman et al., 2011). The cognitive dynamics of the decision process can be inferred from the mouse trajectories, repeat patterns, velocity, and switches in direction. McKinstry et al. (2008) used a mouse-tracking experiment to demonstrate the relationship between cognitive dynamics and arm movements. They asked participants to indicate the truthfulness of a statement by moving their mouse to a “yes” or “no” location on a computer monitor. Greater uncertainty was associated with broader distributions, greater absolute curvatures, and lower peak velocities of mouse movements. In other words, when a participant was relatively uncertain about a statement’s truthfulness, their mouse movement tended to be slower and wander more. In this context, arm movement reflects continuously updated motor commands during a dynamic, high-level decision-making process (McKinstry et al., 2008).

Several recent papers have used mouse tracking to inform our understanding of risky online behavior. Iuga et al. (2016) used mouse-tracking heat maps to hypothesize about the potential lack of mouse focus in areas of interest corresponding to security indicators. In addition, Kelley and Bertenthal (2016a) analyzed participants’ mouse trajectories to assess how websites were searched before a decision was made to login or back out of a specific website. The results revealed that mouse trajectories differed as a function of the encryption level specified in the browser chrome and also as a function of whether the domain names were correct or spoofed. In essence, mouse trajectories were used as a second set of dependent variables in this research and covaried with the accuracy of the participants’ responses.

It remains an empirical question as to whether this real-time information about user behavior could also serve as a predictor of threat detection by users. The present study compares the accuracy of naïve, survey-based, and real-time measures models in predicting risky online behavior during phishing attacks. Mouse tracking is used as a relatively novel yet highly accessible measure of dynamic user behavior during phishing scenarios. We apply this technique while users visit different websites and decide whether or not to login by moving a mouse to one of two different locations depending on whether they want to login or back out.

The experiment was designed to protect users from any real online risks while web-surfing, but this compromises the ecological validity of the study because freedom from risk reduces cognitive load and stress while making decisions. As a consequence, we introduced rewards and penalties such that users experienced the risks and advantages of logging in to various websites. Participants were motivated to move quickly through the experiment in order maximize their payout, and were penalized for incorrectly responding to spoof and non-spoof websites. Thus, the experiment was designed to include time pressure often encountered in everyday life while also making it “risky” for participants to incorrectly respond to spoof and non-spoof websites. After completing the website identification task, participants completed a questionnaire assessing their practical and technical security knowledge, knowledge of security indicators, and degree of familiarity with the websites simulated during the experiment. Given our previous research (Kelley and Bertenthal, 2016a,b) indicating that participants often ignore security indicators, it was hypothesized that participants would not perform better than chance at identifying insecure, or “spoof,” websites and would demonstrate a bias toward logging in to websites. It was also expected that a model utilizing knowledge of security indicators and website familiarity would be more predictive of risky online behavior than a two-factor model including only information specified by the experimental design (i.e., spoof vs. non-spoof websites and variations in security indicators). Lastly, it was hypothesized that a model utilizing real-time indicators of mouse tracking behavior would provide the best predictive value of risky online behavior during potential phishing attacks.

## Materials and Methods

### Participants

Participants were recruited from Amazon’s Mechanical Turk using a protocol approved by Indiana University’s Internal Review Board. Participants were eligible to take part in the study if they were 18 years or older, fluent in English, and utilized a Mozilla Firefox browser. The initial sample consisted of 214 participants. Before data analysis, 41 participants were excluded from the study for not completing the task. An additional 50 participants were excluded from analysis for only making one type of response to all websites (e.g., for all websites the participant chose to press ‘login’). The final sample included data from 123 participants. Forty-one percent of participants in the final sample were female, and participants had a median age of 30 (95% UI = 18, 53).

For completeness we evaluated the models with both the final sample and the larger sample of all participants that completed the study (*N* = 173). The full sample of participants that completed the task was similar to the reduced sample. Forty-two percent of participants were female, and the median age was also 30 (95% UI = 18, 54).

### Stimuli

**FIGURE 1:**
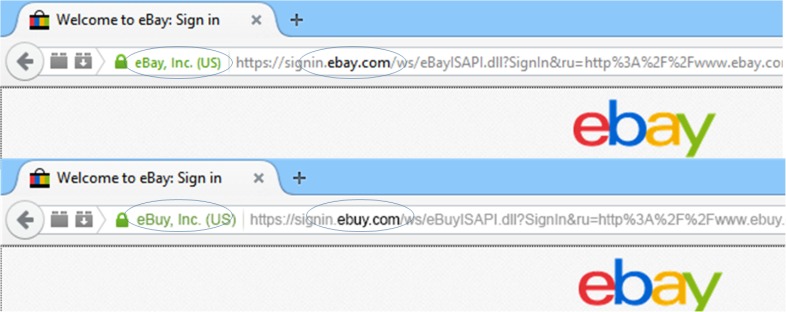
Example website manipulations. The top image depicts a legitimate website, with both a valid URL in bold and extended validation (EV) certificate in green text. The bottom image depicts a sample spoof website with modified URL and EV certificate (Kelley and Bertenthal, 2016b).

Website images were consistent with those presented on a Mozilla Firefox browser, and participants were required to use Firefox throughout the task. Adobe Photoshop CS6 13.0.1 ×64 was used to create spoof and non-spoof versions of six different websites at 1920 × 1080 pixel resolution. There was one spoof and one non-spoof website for each of the following three authentication levels: (1) Extended validation (EV) displayed a green lock and “https.” This configuration indicates that the website has had extended vetting by a certificate authority and full encryption, respectively, (2) Standard validation (SV) displayed a gray lock and “https” indicating domain validation only and full encryption, and (3) Partial encryption (PE) displayed a triangle with exclamation mark, indicating that some (unknown) elements of the website were encrypted. All stimulus configurations were consistent with those that are technically feasible on the Internet. For example, the URL bar could not list a URL beginning with “https” and display a partial encryption indicator. Similarly, spoof conditions displaying an extended validation certificate could not point to a non-spoofed entity (i.e., ebuy.com’s extended validation certificate would list ebuy as its validated entity, but it could not list eBay as the verified entity). Matched versions of the spoof and non-spoof websites were created by altering one letter of the domain name for the spoof website’s URL (see **Figure 1**), and, if necessary, manipulating the owner of the extended validation certificate. HTML image maps were used to simulate functionality in the websites’ login and back buttons on the simulated browser. The stimuli were presented to participants in a pop-up window with the Mozilla Firefox browser disabled to minimize confusion between the simulated websites’ browser chrome and the participants’ actual browser. The disabled browser also prevented participants from reloading pages or navigating backward or forward through the experimental stimuli.

### Procedure

Participants were presented with a novel two-alternative forced-choice paradigm where they were asked to decide whether or not to login to a series of websites based on their judgment of the website’s security. Participants received $2.00 for completing the study and received bonus pay for completing the task as quickly and accurately as possible. Each participant had the opportunity to earn $8.00 in bonus pay, but this bonus pay decreased linearly with the time it took participants to complete the task. Incorrect responses resulted in time penalties. Logging into an insecure website resulted in a 10 s penalty, while clicking on the back button for a legitimate website resulted in a 20 s penalty. Differences in penalty time were driven by pilot tests which showed that, given the fixed location of the simulated back button, participants could respond with a back response much quicker than a login response.

The average bonus pay received was $2.34 (95% UI = $0.00, $4.83). The average time it took to respond per site was 9.04 s (95% UI = 3.52, 27.2), and the mean total time to complete the task was 54.2 s (95% UI = 25.1, 99.1). Bayesian correlations showed no effect of total time on accuracy (β = -0.0002; 95% UI = -0.002, 0.001) nor an effect of mean time per site on accuracy (β = 0.002; 95% UI = -0.003, 0.008). Unsurprisingly, a participants’ total time had a negative effect on their total bonus pay with each additional second spent reducing the bonus pay by about $0.04 (β = -0.04; 95% UI = -0.05, -0.03).

On each trial, participants were first presented with a “start trial” screen containing only a “start trial” button. On the “start trial” screen no timers were running. Once participants clicked on the “start trial” button, they were presented with the Homepage of a simulated website. They then had to click on an active login link, which advanced the experiment to the second page where participants had to choose between logging into the website by clicking either the “sign in” or “back” button. If participants decided that the site was secure, they clicked on the “sign in” button, but if they decided that the site was insecure, they clicked on the “back” button. Presenting the Homepage of the website followed by the login page ensured that all participants would begin each trial with their mouse curser at the same location. The locations of the “start trial” and “back” buttons were constant across all the trials, but the locations of the login link and “sign in” buttons varied across trials.

Once participants decided whether the website was secure or malicious and clicked on the corresponding button (i.e., login or back, respectively), the screen advanced to the next “start trial” screen. After finishing six trials, participants completed a survey including demographic (e.g., age, gender), security knowledge, and website familiarity questions. Self-reported use of security indicators was computed from the number of correct and incorrect security indicators identified in the survey (#correct indicators+1/#incorrect indicators+1) resulting in an indicator score ranging from 0.2 to 4.0. Security knowledge was assessed through 10 multiple-choice questions on formal knowledge of information security (e.g., what type of math is used in the RSA algorithm) resulting in a score from 0 (none correct) to 1 (all correct) (Ben-Asher and Gonzalez, 2015). The website familiarity questions asked participants to indicate their level of familiarity with the websites that were presented on the six trials. Participants rated their familiarity with a given website using a five-point Likert scale ranging from *1* = not familiar to *5* = very familiar. The survey included additional questions focusing on self-reported technical experience, but they were not used in this experiment because the security knowledge questions provided a single continuous predictor of technical experience rather than numerous ordinal or categorical predictors.

### Design

Each participant was presented with three spoof and three non-spoof trials, hereafter referred to as “site type.” Within each of these two conditions, there was one website for each of the following three authentication level conditions: (1) Extended Validation (EV), (2) Standard Validation (SV), and (3) Partial Encryption (PE). Stimuli were presented to participants in a counter-balanced design with half of the participants viewing the same three spoof websites and the same three non-spoof websites in random order, and the other half of the participants viewing the reverse (i.e., spoof version of the non-spoof websites and vice versa).

### Models and Analyses

The present research examines the added value of including real-time measures of the decision-making process in predicting the identification of phishing websites. To this end, three different models of participants’ ability to identify malicious websites were compared. All three models were hierarchical Bayesian logistic regression models using participants as partially pooled group-level predictors with individuals’ ability to correctly identify malicious websites (i.e., accuracy) as the dependent measure. Each model used robust, weakly informed Student-*t* priors with three degrees of freedom. Coefficient priors had a scale of five, while priors for the intercepts had a scale of 10 (Gelman et al., 2008; Ghosh et al., 2017).

The first model, dubbed “two-factor model,” tested participants’ accuracy as a function of the experimental manipulations (i.e., site type and authentication level) as well as the group-level predictors. The second, “survey-based” model, tested participants’ security knowledge and their familiarity with the websites in addition to the predictors included in the first model. The third, “real-time measures” model, included the previously mentioned predictors, in addition to three real-time measures of mouse movement: area under the curve (AUC), sample entropy (SE), and response time (RT) to login or back out of the website. Descriptive statistics for each individual predictor are found in **Table 1**. Any differences between the models analysis using the selected sample and the full sample of participants that completed the study are noted in the relevant model analysis section.

**Table 1:** Correlation and descriptive statistics (*N* = 736).

Variables	Accuracy	Knowledge	Familiarity	AUC	SE	RT
Accuracy	–					
Knowledge	0.20^∗∗∗^	–				
Familiarity	0.11^∗∗^	-0.01	–			
AUC	-0.07	0.02	-0.11^∗∗^	–		
SE	-0.07	0.07	-0.00	0.28^∗∗∗^	–	
RT (s)	0.04	0.04	-0.07	0.12^∗∗^	0.00	–
*M*	0.64	0.52	3.17	1.31	-2.34	8.96
*SD*	0.48	0.25	1.60	1.29	0.65	0.51
Range	0 – 1	0.1 – 1.0	1 – 5	-2.34 – 4.67	-5.58 – -0.72	7.74 – 11.37

*^∗∗∗^p < 0.001, ^∗∗^p < 0.01, ^∗^p < 0.05.*

Interpretations of the regression coefficients can be done in the standard manner of adding the coefficients together starting from the intercept. For example, each model intercept corresponds to the average accuracy in the non-spoof, standard validation condition, with all continuous measures centered at 0. To look at the difference in accuracy in the non-spoof/spoof manipulation, one would compare β_intercept_ and β_intercept_ + β_spoof_. Since the parameter estimates are on the logistic scale, one can use the inverse logistic function to convert the estimates back to a probability. Since we are using Bayesian analysis, another way to investigate the parameter estimates is to sample directly from the posterior distribution given the parameter values of interest. Sampling the posterior is often easier than trying to calculate all the values in the regression equation, and is used to generate the model figures.

We used Bayesian statistics wherever possible. One of the advantages of using a Bayesian approach is that it gives a broad number of solutions for examining models (Gelman and Rubin, 1995). In particular, a Bayesian analysis provides evidence for a model based on the data, which allows comparison with other models, rather than comparison against a null model (Myung and Pitt, 1997; Rouder et al., 2009; Kass and Raftery, 2015; Morey et al., 2016b). Bayesian analysis also allows us to use proper confidence intervals, while mitigating the problems with multiple comparisons (Gelman et al., 2012; Hoekstra et al., 2014). We call the confidence intervals *uncertainty intervals* to highlight the fact that the interval being reported represents the uncertainty of the estimation; wider distributions mean greater uncertainty about the actual estimation. This makes reading the uncertainty intervals of an estimation straightforward. We use α = 0.05 throughout the paper. Since we have proper uncertainty intervals, an estimated parameter value has (1-α)% chance of falling within the given uncertainty interval (Morey et al., 2016a; Kruschke and Liddell, 2017).

To ascertain the evidence for a given model, we use the expected log-pointwise predictive density (ELPD). As the ELPD, or log-likelihood, in a model increases, it indicates that the model is more probable given the data. ELPD has the advantage over using mean-squared error to evaluate models in that ELPD can be applied to models that are not normally distributed. ELPD also allows models to be compared equivalently and supports continuous model expansion and evaluation rather than specific model selection (Gelman et al., 2013b). ELPD in standard analysis is usually a pointwise estimate, say a maximum likelihood estimate, but, since we have the posterior distributions, we can examine the distribution of ELPD for each set of samples.

In addition to ELPD, we also use posterior predictive checking on the generated contingency tables to assess model accuracy. Model accuracy is the number of true positives and true negatives predicted over the total number of predictions. Due to the small amount of data in our sample we use 10-fold cross-validation to assess model performance with the in-and-out-of-sample data for both ELPD and model accuracy. In-sample data are the data the model is trained on, while out-of-sample data are held out of the training and used to test the model’s fit. While this approach does not completely remove the biased estimate from looking at in-sample predictive accuracy, it somewhat balances the difficulties of evaluating out-of-sample predictions in sparse data and over-estimating the predictive power of a model.

#### Measures

In each model, individual participants’ accuracy was used as a group-level predictor of accuracy. Thus, the models utilize participants’ observed behavior, but treat that behavior as somewhat homogeneous between participants. This allows the models to include any similarities between predictor levels, at the expense of higher variance. Given the few number of trials in each condition, however, this is useful tradeoff to avoid overfitting (Gelman et al., 2013a). In addition, all three models utilized site type (non-spoof/spoof) and authentication level (PE < SV < EV) as predictors of accuracy. In this case site type was a two-level factor and authentication level was treated as an ordinal factor with three levels. Partial encryption contains less authentication information than full encryption, which contains less information than extended validation. The second and third models included survey-based predictors of accuracy (i.e., security knowledge and website familiarity).

Response time, AUC, and SE were added to the third model as real-time predictors of login accuracy. All three of these measures have been used to demonstrate the effects of conflicting options on final decisions (Freeman et al., 2008). Rather than examining the end product of the decision, both AUC and SE measure different characteristics of mouse behavior leading to the final decision, while RT is a measure of the time it takes an individual to make their final response. In this case, RT was calculated as the time between the login page first appearing and the time when participants clicked a final response.

**FIGURE 2:**
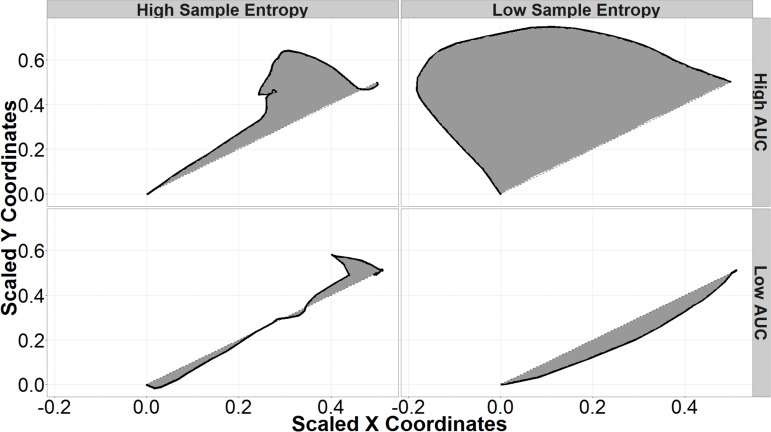
Sample entropy (SE) and area under the curve (AUC) of sample mouse trajectories. The black lines represent Mouse trajectories and shaded areas represent the AUC. Trajectories with high SE (left column) are more variable; low SE trajectories are smooth curves (right column). High AUC trajectories (top row) deviate further from a hypothetical straight-line trajectory than those with low AUC (bottom row).

To account for different screen sizes and resolutions, each mouse trajectory was normalized by scaling the straight-line response from the click to load the final login page and the final response click to a unit vector. The optimal responses were then rotated to fall along a 45° angle from origin for login responses and a 135° angle for back response. This scales all trajectories to the same state space beginning at the origin with the distance of the optimal path being 1, and allows trajectories to be compared on the same scale. Example normalized login responses are found in **Figure 2**.

Area under the curve is the area formed by connecting the actual mouse trajectory and a straight-line path between the start of the trajectory and its end. Trajectories with high AUC deviate further from straight-line paths than do trajectories with low AUC (**Figure 2**). Previous research indicates that higher AUC corresponds to higher uncertainty in each response, while lower AUC corresponds to more certain responses (Dale et al., 2007; Freeman et al., 2010, 2011). In the current research, a participant may start out at the top right of the screen and move in an arc toward the back button (top left of the screen) before curving the mouse toward the final login response. Given that that the presence of security indicators has previously been shown to affect participants’ AUC when responding to phishing websites, this research attempts to use AUC as an independent predictor to better understand the decision-making process in different situations (Kelley and Bertenthal, 2016b).

Sample entropy is another tool for examining the dynamics of mouse trajectories during decision making and can be seen as a “complexity index” (Richman and Moorman, 2000). Trajectories that are more complex or irregular have higher sample entropies, while less complex trajectories have low SE and appear as smooth curves in this application (**Figure 2**). Since standard SE is sensitive to input parameters, multi-scale sample entropy (MSSE) was used as a more robust measure that accounts for different radii and dimensional encodings (Yentes et al., 2013). SE was calculated on the distance of each point from the final response. This takes into account both x and y motion. The final SE was taken as the MSSE calculated using radii from 0.01 of the sample standard deviation (a very local measure) to 0.25 of the sample standard deviation (a more global measure), dimensional encodings between 2 and 8, and a Theiler Window based on autocorrelation as described in Theiler’s work on the dimensions of strange attractors (Theiler, 1986).

As with AUC, SE has been used to observe the effects of uncertainty between competing views in decision-making tasks (Dale et al., 2007; McKinstry et al., 2008). In this research, increases in SE could correspond to increases in uncertainty in identifying a spoof website. Higher SE would correspond to more switches in direction. For example, a participant with higher uncertainty between choices may show behavior toward login, then move toward the back button, before finally switching back to click on the login button. Including SE as an independent measure allows for the exploration of different types of correct or incorrect responses. For example, there may be conditions that interact with SE to create low SE errors—incorrect responses because the participant is too certain—and high SE errors—incorrect responses due to too much uncertainty. **Figure 2** summarizes the types of mouse trajectories produced by different combinations of SE and AUC.

## Results

### Accuracy

Mean accuracy in identifying spoof and non-spoof websites was 0.64 (95% UI = 0.17, 1.00). The finding that mean accuracy was above 0.50 was due primarily to high accuracy in the non-spoof condition (μ = 0.79, 95% UI = 0.33, 1.00); accuracy in the spoof condition was at chance (μ = 0.49, 95% UI = 0.00, 1.00). The results also revealed a strong bias to login regardless of the security indicator.

**FIGURE 3:**
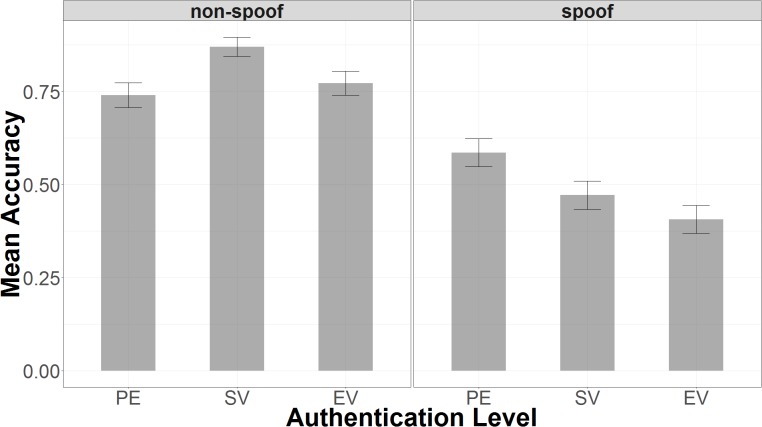
Accuracy plotted as a function of authentication level and non-spoof/spoof condition.

Accuracy in the spoof condition decreased as a function of encryption level (PE < SV < EV), and it also varied non-monotonically with encryption in the non-spoof condition (see **Figure 3**). In the spoof condition, there was nearly 60% accuracy with partial encryption (μ = 0.59, 95% UI = 0.00, 1.00), but accuracy declined with standard validation (μ = 0.47, 95% UI = 0.00, 1.00), and declined even further with extended validation (μ = 0.41, 95% UI = 0.00, 1.00). This finding suggests that false confidence in the security of the website might increase with the level of encryption. Bayesian *t*-tests showed that responses in extended validation were credibly less accurate than both standard validation (μ_EV -SV_ = -0.08, 95% UI = -0.15, -0.01) and partial encryption (μ_EV -PE_ = -0.21, 95% UI = -0.28, -0.15). Extended validation responses were also found to be credibly less accurate than partial encryption responses (μ_SV -PE_ = -0.14, 95% UI = -0.20, -0.06). Note that a credible difference (similar to a significant difference in frequentist statistics) is concluded when the 95% uncertainty interval (UI) does not include an effect size of 0.

In the non-spoof condition, accuracy was highest with standard validation (μ = 0.87, 95% UI = 0.00, 1.00), but lower for partial encryption (μ = 0.74, 95% UI = 0.00, 1.00) and extended validation (μ = 0.77, *SD* = 95% UI = 0.00, 1.00). Bayesian *t*-tests revealed that response accuracy was reliably greater with standard validation as opposed to either partial encryption (μ_SV -PE_ = 0.13, 95% UI = 0.09, 0.18) or extended validation (μ_SV -EV_ = 0.11, 95% UI = 0.06, 0.15). There was no credible difference between partial encryption and extended validation response accuracy (μ_EV -PE_ = 0.005, 95% UI = -0.05, 0.06).

### Security Knowledge and Website Familiarity

On average, participants answered 49.8% of the questions assessing security knowledge correctly (*SD* = 25%). Increased security knowledge was associated with increased accuracy in identifying websites across all trials [*r*(736) = 0.2, *p* < 0.001]. This was true in both spoof [*r*(367) = 0.25, *p* < 0.0001] and non-spoof conditions [*r*(367) = 0.18, *p* < 0.001].

Familiarity with a given website was also associated with greater accuracy when identifying websites. This measure ranged between 1 (*not familiar*) and 5 (*very familiar*) and averaged (μ = 3.17, *SD* = 1.60) across all trials. Greater familiarity with a given website covaried with accuracy *r*(736) = 0.11, *p* < 0.01]. This relation was significant in both the spoof [*r*(367) = 0.11, *p* < 0.05] and non-spoof conditions [*r*(367) = 0.17, *p* < 0.001].

Security knowledge and website familiarity were not significantly correlated with one another [*r*(736) = -0.01, *p* < 0.83]. Self-reported use of security indicators was not found to be associated with accuracy as a function of either authentication level or non-spoof/spoof condition. Since there was no correlation between indicator score and accuracy, this score was not used as a predictor in the tested models. By contrast, both security knowledge and familiarity were found to be correlated with increases in accuracy for at least one of the two independent variables, and thus they were both included in the survey-based model and real-time measures model.

### Real-Time Measures

Real-time measures were associated with accuracy, but this relation was modulated by the non-spoof versus spoof condition. AUC was not correlated with accuracy collapsed across conditions [*r*(736) = -0.07, *p* = 0.06; *M*_AUC_ = 8.09, *SD*_AUC_ = 12.09] or in the non-spoof condition [*r*(367) = 0.08, *p* = 0.14]. In the spoof condition, however, increased AUC was linearly associated with decreased accuracy, [*r*(367) = -0.26, *p* < 0.001]. Even though AUC was not correlated with accuracy collapsed across conditions, higher AUC was correlated with a higher likelihood to login, [*r*(736) = 0.20, *p* < 0.001]. This suggests that the covariation between AUC and accuracy might be related to the screen locations of the back and login buttons, because the latter button shifted somewhat from one trial to the next.

Sample entropy was not correlated with overall accuracy [*r*(736) = -0.07, *p* = 0.08; *M*_SE_ = 0.12, *SD*_SE_ = 0.08] or accuracy in the spoof condition [*r*(367) = 0.01, *p* = 0.90]. By contrast, higher SE in the non-spoof condition was associated with lower accuracy, [*r*(367) = -0.13, *p* = 0.01].

Like both AUC and SE, there was no correlation between RT and overall accuracy, *r*(736) = 0.04, *p* = 0.22; *M*_RT_ = 9.04, *SD*_RT_ = 6.56. Unlike AUC and SE, the lack of relationship between RT and accuracy across all trials was due to offsetting relationships in the two conditions. Increases in RT (slower responses) were associated with reductions in accuracy in the non-spoof condition [*r*(367) = -0.1, *p* < 0.05], but higher accuracy in the spoof condition [*r*(367) = 0.18, *p* < 0.001].

### Correlations Between Predictors

As mentioned above, security knowledge and website familiarity were not significantly correlated. Website familiarity was, however, negatively correlated with AUC, *r*(736) = -0.11, *p* < 0.01. AUC was also positively correlated with SE [*r*(736) = 0.28, *p* < 0.001] and RTs [*r*(736) = 0.12, *p* = 0.001]. This may occur as larger AUCs likely take more time and involve larger path deviations to execute. As shown in **Table 1**, SE was not associated with RT, *r*(736) = 0.002, *p* = 0.94. It might have been expected that higher security knowledge or higher website familiarity might have resulted in faster RT, but this was not observed, *p* > 0.05 (see **Table 1**).

In order to capture the correlations between AUC, SE, and RT, the survey-based and real-time measures models included three-way interactions between predictors. Including all three-way interactions in the survey-based measures model also preserved the changes in correlation between the spoof and non-spoof conditions for both the security knowledge and website familiarity predictors.

### Two-Factor Model

The two-factor model captured the effects of both the non-spoof/spoof manipulation and the authentication manipulation in addition to individual variation among participants. Examining the posterior distributions shows that this model was relatively accurate in capturing the observed data. Sampling from the posterior distribution showed that the model predicted accuracy in the non-spoof condition (μ = 0.83, 95% UI = 0.49, 0.99), but was essentially random in the spoof condition (μ = 0.48, 95% UI = 0.11, 0.87). The model predicted that a non-spoof website would be correctly identified 33% more often than a spoof website and that this difference is credible, despite the high variance—most of which is found in responses to the spoof condition (μ_diff_ = 0.33, 95% UI = 0.11, 0.46).

The two-factor model also captured the relationship between authentication level and non-spoof/spoof condition. In the non-spoof condition, there was no difference between authentication levels in terms of accuracy: extended validation had the highest accuracy (μ = 0.81, 95% UI = 0.44, 0.98), followed by standard validation (μ = 0.79, 95% UI = 0.42, 0.98) and partial encryption (μ = 0.78, 95% UI = 0.38, 0.98). In the spoof condition, increases in authentication level corresponded to decreases in accuracy: partial encryption (μ = 0.58, 95% UI = 0.17, 0.92) was associated with greater accuracy than standard validation (μ = 0.49, 95% UI = 0.12, 0.88), which was, in turn, associated with greater accuracy than extended validation (μ = 0.40, 95% UI = 0.08, 0.83). Because of the linear ordering of encryption levels, each increase in authentication level (i.e., PE→SV and SV→EV) led to a decrease in accuracy of approximately 9% (μ_diff_ = 0.09, 95% UI = 0.02, 0.16).

As **Table 2** shows, the two-factor model captured the effects of the experimental variables, but it did not reveal any specific information about how these decisions were processed. More information about aspects of user decision making must be evaluated in order to better understand the conditions under which security cues fail.

**Table 2:** Two-factor model coefficient estimates from those estimated to have credible effects different than zero.

Coefficient	Est. β (95% UI)	Est. error	Eff. samples	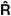
Population-level effects				
Intercept	1.63 (1.29, 2.00)	0.19	20,000	1
Spoof	-1.69 (-2.08, -1.31)	0.20	20,000	1
Spoof × authentication	-0.56 (-1.01, -0.12)	0.31	20,000	1
Group-level effects				
*SD* (intercept)	1.03 (0.73, 1.37)	0.16	6,494	1

### Survey-Based Model

Adding both security knowledge and website familiarity to the model improves our understanding of the decision-making process. As shown in **Table 3**, the survey-based model indicated that both security knowledge and familiarity contributed to susceptibility of phishing attacks. In particular, increased security knowledge (β = 1.15, 95% UI = 0.40, 1.91) and website familiarity (β = 1.15, 95% UI = 0.47, 1.85) were associated with better ability to discriminate between spoof and non-spoof websites.

**Table 3:** Survey-based model coefficients with credible effects different than zero.

Coefficient	Est. β (95% UI)	Est. error	Eff. samples	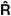
Intercept	1.88 (1.48, 2.30)	0.21	10,291	1
Spoof	-1.93 (-2.38, -1.49)	0.23	20,000	1
Knowledge	1.15 (0.40, 1.91)	0.38	10,919	1
Familiarity	1.15 (0.47, 1.85)	0.35	12,123	1
Spoof × authentication	-0.83 (-1.35, -0.33)	0.26	13,537	1
Familiarity × authentication	1.09 (0.30, 1.89)	0.40	14,734	1
Group-level effects				
*SD* (intercept)	0.88 (0.55, 1.22)	0.17	7,374	1

While security knowledge was beneficial for all authentication levels, the two-way interaction between website familiarity and authentication level revealed that website familiarity affected accuracy in standard and extended validation, but not partial encryption conditions. Website familiarity was not predictive of accuracy in partial encryption (μ = 0.06, 95% UI = -0.91, 1.01). In the case of standard validation, the normal effects of familiarity were present (μ = 1.15, 95% UI = 0.47, 1.85), however, when an extended validation certificate was present, the website familiarity greatly increased the ability to identify a spoofed website (μ = 2.23, 95% UI = 1.15, 3.39).

The interaction between spoof and authentication shows the same effects as the previous model. Authentication is not predictive of accuracy in the non-spoof condition, but, in the spoof condition, increases in authentication level reduce accuracy (see **Table 3**).

### Real-Time Measures Model

The addition of real-time measures increases model complexity, but reveals that these measures are significant predictors of accuracy, while largely leaving the results from the previous models unchanged. **Table 4** reveals that the effects and directions of the previous models remain credible predictors of accuracy, with slightly different estimated coefficients due to the added predictors. Non-spoof trials are more accurate than spoof trials, and increases in security knowledge and website familiarity improve accuracy.

**Table 4:** Real-time measures model coefficients with credible effects different than zero.

Coefficient	Est. β (95% UI)	Est. error	Eff. samples	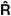
Intercept	2.18 (1.67, 2.75)	0.27	11,079	1
Spoof	-2.43 (-3.07, -1.84)	0.31	12,451	1
Authentication	0.74 (0.20, 1.31)	0.28	13,090	1
Knowledge	2.12 (1.13, 3.20)	0.53	12,074	1
Familiarity	1.10 (0.26, 1.99)	0.44	14,203	1
AUC	1.57 (0.55, 2.63)	0.53	12,973	1
SE	-1.47 (-2.34, -0.64)	0.43	15,560	1
Spoof × authentication	-1.40 (-2.11, -0.73)	0.35	13,581	1
Spoof × AUC	-3.90 (-5.19, -2.64)	0.65	14,241	1
Spoof × SE	2.01 (0.92, 3.15)	0.57	20,000	1
Spoof × RT	1.66 (0.46, 2.85)	0.61	14,821	1
Authentication × AUC	1.73 (0.52, 2.97)	0.62	13,628	1
Knowledge × SE	2.35 (0.66, 4.10)	0.88	20,000	1
AUC × SE	1.60 (0.00, 3.29)	0.84	20,000	1
SE × RT	-2.69 (-4.50, -0.97)	0.90	20,000	1
Spoof × authentication × AUC	-2.26 (-3.83, -0.73)	0.79	14,023	1
Spoof × AUC × SE	-3.25 (-5.52, -1.09)	1.13	20,000	1
Authentication × Knowledge × AUC	1.82 (0.29, 3.37)	0.78	20,000	1
Authentication × Knowledge × SE	-1.54 (-3.02, -0.09)	0.75	20,000	1
AUC × SE × RT^∗^	1.98 (0.14, 3.93)	0.96	20,000	1
Group-level effects				
*SD* (intercept)	0.85 (0.33, 1.31)	0.24	4,313	1

*^∗^The interaction between AUC, sample entropy, and response time, was only credible when the model used the full population that completed the study (N = 173) as opposed to the selected population (N = 123).*

There are, however, two major changes from the survey-based model due to the added predictors. First, the interaction between authentication and familiarity is gone. Second, authentication level becomes an important predictor of accuracy in both non-spoof and spoof conditions, rather than just the spoof condition, as it was in the survey-based model.

#### Response Time

**FIGURE 4:**
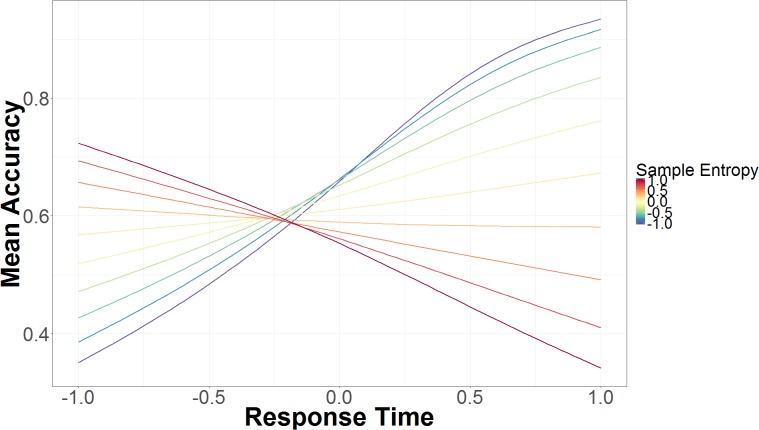
Mean accuracy based on mouse trajectory SE and RT. The data on the x-axis are normalized and range from 2 *SD* below the mean (-1.00) to 2 *SD* above the mean (+1.00).

The interaction between RT and non-spoof/spoof conditions shows that RT is not predictive of accuracy in the non-spoof condition (*B* = -0.16, 95% UI = -1.19, 0.89), but in the spoof condition longer RTs are associated with higher accuracy (*B* = 1.66, 95% UI = 0.46, 2.88). As seen in **Figure 4**, RT also interacts with SE. On the right side of the figure, slower RT paired with lower SE lead to greater accuracy, but as SE increases, accuracy decreases rapidly. Faster RT, seen on the left side of the figure, and lower SE were associated with lower accuracy. As SE increases, so does accuracy, though SE affects accuracy to a lesser extent in fast responses than in slower responses.

Using the full sample introduced another interaction between RT, SE, and AUC. Given this interaction between three continuous variables, we describe the results qualitatively. When RT was average or slower and AUC was average or lower, an increase in SE was associated with reduced accuracy. As AUC increased from low AUC (-1 *SD*) to average AUC, the association between SE and inaccuracy decreased. As RT increased from average RT to slower RT (+1 *SD*), the effect between SE and lack of accuracy increased. When RT was fast (-1 *SD*) or when there was a large amount of AUC (+1 *SD*), there was no association between SE and accuracy. When RT was average and SE was average or high (+1 *SD*), increased AUC was associated with greater response accuracy. Increases in AUC were also associated with better accuracy when RT was slow (+1 *SD*) and SE was high (+1 *SD*). As SE increased, increases in AUC led to greater increases in accuracy. As AUC decreased from average to low (-1 *SD*), RT was associated with accuracy, but SE modulated this association. Decreases in AUC, increased the effects of SE on the association of RT to accuracy. When SE was low (-1 *SD*), increases in RT were associated with increases in accuracy. When SE was high (+1 *SD*), increases in RT were associated with decreases in accuracy.

#### Sample Entropy

**FIGURE 5:**
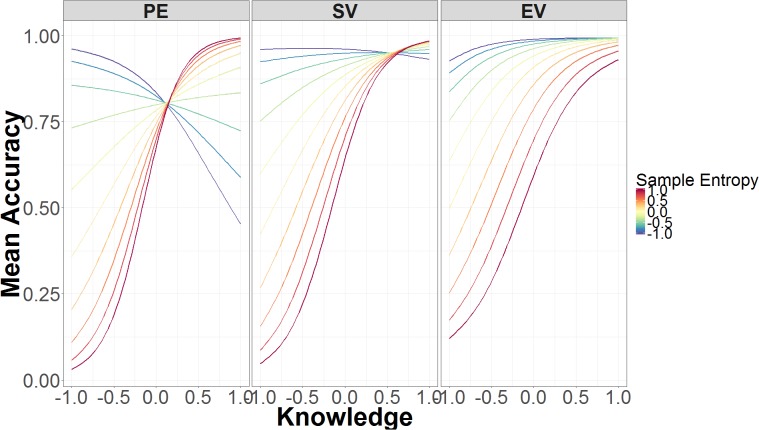
Mean accuracy as a function of SE, security knowledge, and authentication level. The data on the x-axis are normalized and range from 2 *SD* below the mean (-1.00) to 2 *SD* above the mean (+1.00).

Sample entropy is a credible predictor of accuracy in both spoof and non-spoof conditions. Higher SE leads to greater inaccuracy in identifying non-spoof sites (*B* = -1.47, 95% UI = -2.34, -0.64), but leads to greater accuracy in identifying spoof sites (*B* = 2.01, 95% UI = 0.92, 3.15). SE is also involved in a two-way interaction with security knowledge, which is part of the three-way interaction between SE, authentication level, and knowledge. **Figure 5** depicts the interactions between SE, security knowledge, and authentication level. In the standard validation condition (middle panel), low security knowledge and low SE are predictive of high accuracy, whereas higher levels of SE are predictive of low accuracy. As knowledge increases, the predictive power of SE decreases, because accuracy is high regardless of SE.

The relation between SE and security knowledge is very similar in the extended validation condition (**Figure 5** right panel). Just as in standard validation, low levels of knowledge and low SE are associated with higher accuracy, while low levels of knowledge and high SE lead to poorer accuracy. With high knowledge, there is little difference in performance between responses that have high or low SE.

Unlike the preceding two conditions, SE in the partial encryption condition varies at both low and high levels of security knowledge (**Figure 5** left panel). Low knowledge and low SE are still associated with higher accuracy. By contrast, high knowledge and low SE leads to lower accuracy, whereas high knowledge and high SE leads to higher accuracy. As such, the effects of SE are flipped in the low- compared to high-knowledge conditions.

**FIGURE 6:**
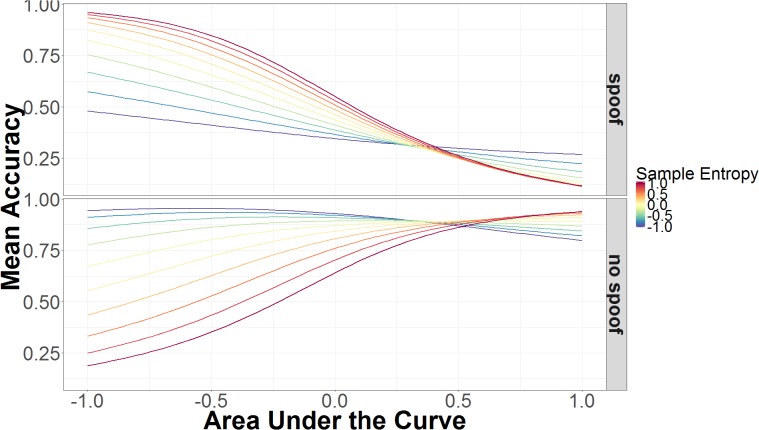
Mean accuracy as a function of AUC and SE in spoof and non-spoof conditions. The data on the x-axis are normalized and range from 2 *SD* below the mean (-1.00) to 2 *SD* above the mean (+1.00).

Sample entropy was also involved in a three-way interaction involving AUC and non-spoof/spoof conditions. As can be seen in **Figure 6**, accuracy increases as AUC increases, but this is modulated by SE in opposite directions for non-spoof and spoof conditions. When AUC is low, high SE is associated with accurate responses in the spoof condition and inaccurate responses in the non-spoof condition, while low SE is associated with less accurate responses in the spoof condition and accurate responses in the non-spoof condition. When AUC is high, these interactions are for the most part canceled and accuracy is primarily determined by the AUC: High AUC results in low accuracy in the spoof condition and high accuracy in the non-spoof condition.

#### Area Under the Curve

As is suggested in the previous section, AUC is another credible predictor of accuracy (β = 1.57, 95% UI = 0.55, 2.63), but it also interacts other predictors in this model. In addition to the three-way interaction between SE and non-spoof/spoof (discussed above), AUC is also involved in two additional three-way interactions. The first interaction involves AUC, authentication level, and non-spoof/spoof. This three-way interaction includes the two-way interactions between AUC and authentication level, and AUC and non-spoof/spoof. The second three-way interaction involves AUC, authentication level, and security knowledge.

**FIGURE 7:**
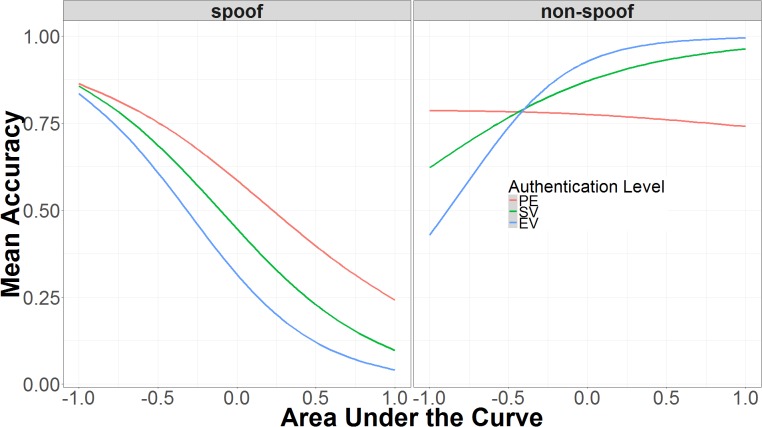
Interaction of AUC, authentication level, and spoof/non-spoof on identifying spoof or non-spoof site. The data on the x-axis are normalized and range from 2 *SD* below the mean (-1.00) to 2 *SD* above the mean (+1.00).

The first three-way interaction is the more straightforward, because it involves two categorical predictors (authentication and non-spoof/spoof) and only one continuous predictor (AUC). As shown in **Figure 7**, in the spoof condition, higher AUC is associated with decreased accuracy, while in the non-spoof condition, higher AUC leads to higher accuracy. The interaction with authentication level makes the relationship between AUC and accuracy more extreme in the non-spoof condition, but has little effect in the spoof condition. In the non-spoof condition, AUC is not predictive in the partial encryption condition. As authentication level increases, the slope of AUC’s effect on accuracy also increases. In the spoof condition, the slope of AUC’s effect on accuracy remains constant across authentication levels, with low AUC associated with high accuracy, and high AUC associated with low accuracy.

**FIGURE 8:**
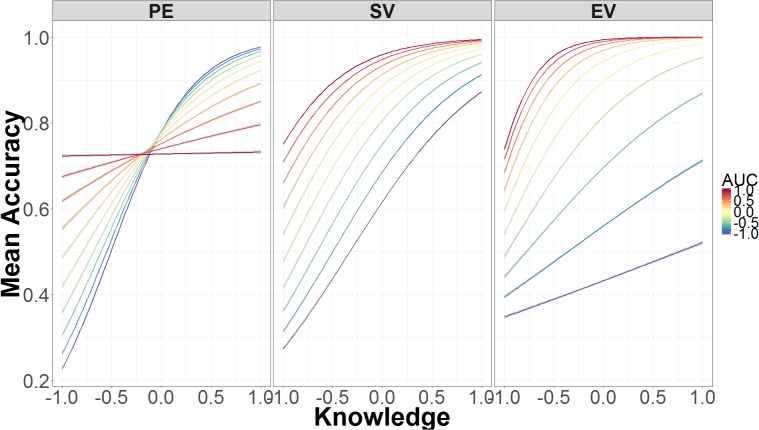
Mean accuracy as a function of the interaction between AUC, security knowledge, and authentication level. The data on the x-axis are normalized and range from 2 *SD* below the mean (-1.00) to 2 *SD* above the mean (+1.00).

**Figure 8** shows how the interaction between AUC, authentication, and knowledge affects accuracy. In the standard validation condition—the middle panel—accuracy is primarily determined by knowledge. As knowledge increases, accuracy increases with roughly the same slope regardless of AUC. This is not the case in the partial encryption (left panel) or extended validation (right panel) conditions. In the partial encryption condition, increases in knowledge still lead to higher accuracy, but decreases in AUC are associated with increases in accuracy. As knowledge declines, decreases in AUC are associated with decreases in accuracy. In other words, the relation between accuracy and AUC flips as a function of knowledge; this is the only condition where this flip occurs. In the extended validation condition, as in the other conditions, accuracy improves with knowledge, but AUC has an additive effect. When AUC is low, there is a minimal increase in accuracy due to knowledge. When AUC is high, accuracy increases much faster as knowledge increases.

### Model Comparison

**FIGURE 9:**
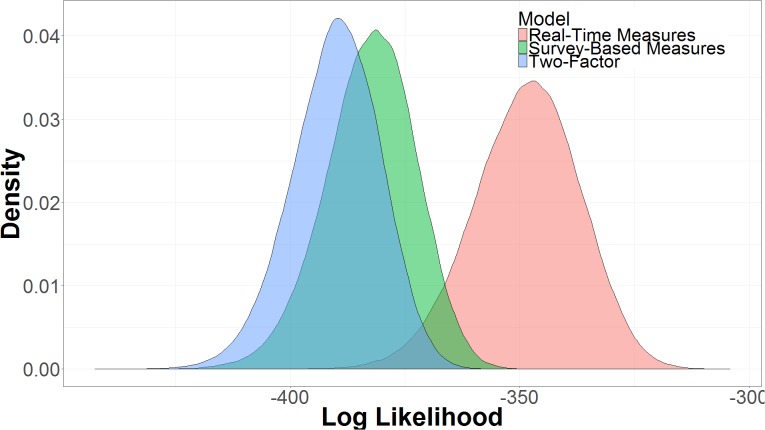
Expected log predictive density taken from posterior samples in 10-fold cross validation.

The three models were compared in terms of their expected log predictive density, sometimes referred to as log likelihood (**Figure 9**). The log likelihood is the log probability of the model producing the observed data, *y*, given the parameter space of the model, 𝜃, or log *p*(*y*|𝜃). Models with more parameters will have more variance in log likelihood. The real-time measures model had a higher log likelihood (μ = -348.56, 95% UI = -372.38, -327.09) than the two-factor model (μ = -390.13, 95% UI = -410.13, -372.41) or the survey-based model (μ = -382.62, 95% UI = -403.26, -364.55). The larger uncertainty interval for the real-time measures model captures the increased uncertainty of performance of a more complex model, but even with the increased complexity, the model using real-time measures is more likely given the data (Myung and Pitt, 1997).

**FIGURE 10:**
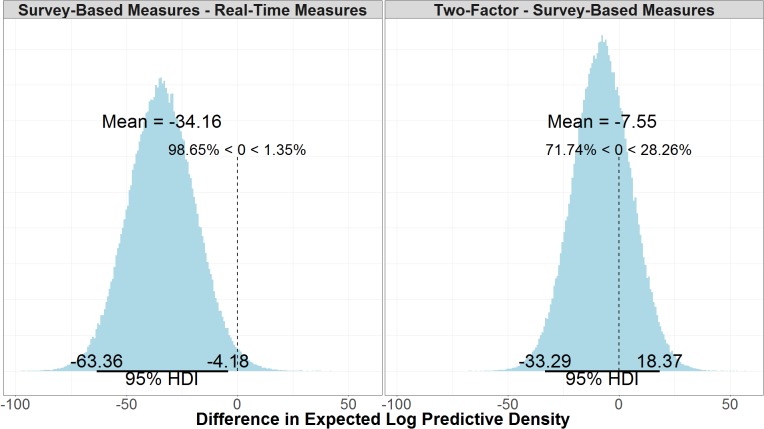
Difference in the expected log predictive density of the real-time measures model and the survey-based model and the survey-based measures model and the two-factor model.

Comparing the log likelihood of the real-time measures model and the survey-based model reveals that there is good certainty that the model with real-time measures is the better model. As seen in **Figure 10**, the real-time measures model is, on average, 34.10 times more likely than the survey-based model (μ = 34.10, 95% UI = 4.18, 63.40). The 95% uncertainty interval shows that there is a 97.5% chance that the real-time measures model is at least 4.18 times more likely than the survey-based model, given the data. We can say that a model (M1) is a certain times better than another model (M2) because log(likelihood_M1_)–log(likelihood_M2_) is equal to likelihood_M1_/likelihood_M2_. **Figure 10**, also shows that, there is some evidence that the survey-based model is a better model than the two-factor model, but it is not conclusive given the uncertainty of the results (μ = 7.51, 95% UI = -18.4, 33.30).

#### Model Accuracy

**FIGURE 11:**
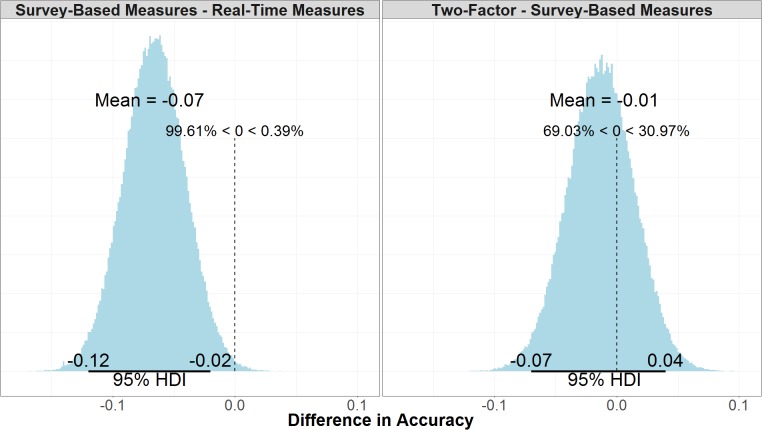
Comparison of accuracy for survey-based and real-time measures models (Left) and two-factor and survey-based models (Right).

Model accuracy refers to the number of correctly predicted true positives and true negatives over the total number of data points predicted, but unlike ELPD it does not consider the model structure or likelihood of future predictions. There is little difference between the accuracy in the two-factor (μ = 0.65, 95% UI = 0.61, 0.69) and survey-based models (μ = 0.67, 95% UI = 0.63, 0.71). As shown in the right panel of **Figure 11**, the mean difference between the two models is negligible (μ = -0.01, 95% UI = -0.07, 0.04). As shown in the left panel of **Figure 11**, the real-time measures model (μ = 0.73, 95% UI = 0.70, 0.77) is credibly more accurate than the survey-based model (μ = -0.07, 95% UI = -0.12, -0.02).

The models’ accuracy was also evaluated in regards to the non-spoof and spoof manipulations to ensure that a model’s composite accuracy was not due solely to excellent performance in one manipulation and random, or poor performance, in the other. In the non-spoof condition, the real-time measures model (μ = 0.78, 95% UI = 0.73, 0.81) was more accurate than both the survey-based model (μ = 0.72, 95% UI = 0.67, 0.76) and the two-factor model (μ = 0.71, 95% UI = 0.66, 0.75). There was a credible difference between non-spoof accuracy in the real-time measures model and the two-factor model (μ = 0.07, 95% UI = 0.01, 0.13). While the difference between the real-time measures model and the survey-based model was less certain (μ = 0.05, 95% UI = -0.003, 0.11), 97% of the samples show that real-time measures model was more accurate than the survey-based model. There was no credible difference between the survey-based model and the two-factor model (μ = 0.01, 95% UI = -0.05, 0.08).

In the spoof condition, the real-time measures model (μ = 0.69, 95% UI = 0.64, 0.74) was again more accurate than the survey-based model (μ = 0.62, 95% UI = 0.56, 0.67) and the two-factor model (μ = 0.60, 95% UI = 0.54, 0.66). The real-time measures model was credibly more accurate than both the survey-based model (μ = 0.08, 95% UI = 0.002, 0.15) and the two-factor model in the spoof condition (μ = 0.09, 95% UI = 0.01, 0.17). All the models predicted non-spoof responses more accurately than spoof responses, but the prediction accuracy of the real-time measures model in the non-spoof condition, was not credibly different than either the survey-based model (μ = 0.03, 95% UI = -0.10, 0.04) or the two-factor model (μ = 0.02, 95% UI = -0.08, 0.05) in the spoof condition.

## Discussion

This study utilized a simulated web-surfing scenario to examine user decision-making under conditions of uncertain website security. The procedure involved a semi-naturalistic framework for examining web behaviors experimentally and ethically. User identification of phishing websites was examined as participants decided whether or not to login or back out of spoof or non-spoof websites with different levels of authentication. Participant accuracy ranged considerably (17–100%). Despite being primed to the possibility of website phishing attacks, participants generally showed a bias for logging in to websites versus backing out of potentially dangerous sites. Overall, accuracy in detecting security threats was 64%, which is nearly the same as the accuracy reported in a similar study involving different attack vectors (Heartfield et al., 2016). As expected, spoof websites were more difficult to correctly identify (49% correct) than non-spoof websites (69% correct).

Three different models for predicting the accuracy of participants’ responses to different websites were compared. Accuracy in identifying spoof and non-spoof websites was best captured using a model that included real-time measures of decision-making, in addition to the two independent variables and survey-based information. The two-factor model incorporated information regarding non-spoof vs. spoof websites as well as the level of authentication. The results demonstrated that a two-factor model captures basic response trends, including a bias to login to websites regardless of their site type and decreased accuracy in identifying spoof websites with increases in authentication. Overall, the two-factor model predicted that participants would be 80% accurate in the non-spoof condition, but at the level of chance in the spoof condition. When compared to the observed data, the two-factor model correctly predicted responses 65% of the time. The relative accuracy of the two-factor model may have been enhanced, in part, due to the response bias to login, which increased the likelihood of responding correctly on all non-spoof trials.

The inclusion of security knowledge and website familiarity improved the accuracy of the survey-based model relative to the two-factor model, but not significantly. Nevertheless, the results indicated that self-reported security knowledge and website familiarity were associated with improved performance in identifying spoof and non-spoof websites. Security knowledge improved website identification accuracy across all authentication levels, while website familiarity resulted in greater accuracy when viewing standard and extended validation websites but not those with partial encryption. Moreover, participants visiting extended validation sites that were spoofed were more likely to login than those visiting partial encryption or standard validation websites. As such, the survey-based model captured more nuanced differences in phishing detection than did the two-factor model.

One interpretation for the interaction between spoof websites and authentication levels is that extended validation increased participants’ confidence in the security of the website, and thus they were more likely to login to malicious websites. The problem with accepting this interpretation is that the addition of real-time measures in the third model reveals that participants’ decision making was more complicated and depended on multiple modulating factors.

It is also noteworthy that security knowledge was systematically related to the correct identification of the websites, but it did not interact with any of the other variables. In many studies of cybersecurity, security knowledge is considered a major factor in explaining individual differences (e.g., Vishwanath et al., 2011; Stembert et al., 2015), but this seemed less true in the current study at least in the context of the survey-based model. Critically, security knowledge was the one variable that was constant across all trials, and thus there was no variability to increase the likelihood of an interaction with other factors. By contrast, the other survey-based measure, website familiarity, varied with each trial. As will be discussed next, the addition of the real-time measures improved significantly the predictive power of the third model, in part because each measure changed continuously between trials as well as within trials.

The addition of the real-time measures revealed that the effects of non-spoof/spoof and authentication-level manipulations, security knowledge, and website familiarity were further modulated by the trajectories and timing of the mouse movements. For example, slower RTs were associated with greater accuracy when responding to spoof websites. By contrast, there was no reliable relationship between RT and threat detection when responding to non-spoof websites. In addition, mouse trajectories with high AUC, or those deviating further from straight-line paths, corresponded to lower accuracy in spoof conditions and higher accuracy in non-spoof conditions. This relationship was further moderated by SE, revealing a complex interplay between experimental factors and real-time behaviors. The relationship between SE and accuracy was also dependent on non-spoof/spoof condition and security knowledge. For standard and extended validation websites, individuals with lower knowledge tended to be less accurate with higher SE, while participants with lower SE tended to be more accurate. By contrast, participants with higher knowledge tended to be more accurate regardless of their SE because the variation became highly constrained with increasing security knowledge. For partial encryption websites, the effects of SE on accuracy were reversed for low and high security knowledge.

The multiple interactions between the real-time measures and the other categorical and continuous variables are difficult to completely decipher as this time. Based on previous research (Kelley and Bertenthal, 2016b), we suggest that AUC is often associated with visual search and will tend to expand when individuals decide to login, because the location of the login button varies from one site to the next. SE is associated with uncertainty in decision making, and this type of variation in mouse movement is observed independent of RT or AUC. Differences in RT were consistent with the distinction between type I vs. type II decision making (Kahneman, 2011). Some of the decisions were very fast and automatic (type I), whereas others were slower and more deliberate and thoughtful (type II). Slower RTs were associated with greater accuracies for spoof but not for non-spoof conditions suggesting that faster or automatic responses were less likely correct, which would have penalized participants in the spoof, but not non-spoof, condition. Currently, these characterizations of the real-time measures are merely heuristic guesses that will require further systematic research to test whether they have merit.

The major finding emerging from this study was that the inclusion of the mouse-tracking measures improved the accuracy of the overall model. The real-time measures model was also more accurate than the survey-based and two-factor models in predicting responses in the spoof condition but not the non-spoof condition. Accuracy improved from 65% for the two-factor model to 67% with the inclusion of the survey information to 73% with inclusion of the real-time measures. One of the potential reasons that the mouse tracking measures increased the accuracy over the survey-based measures is that the mouse trajectories captured the decision-making process as it unfolds over time. Using surveys to assess users’ online behavior assumes that self-reported knowledge or behavior is reflective of real-world decision making, which is not always the case. Knowledge of security indicators, for instance, does not guarantee that users will consistently devote time and attention to browser information prior to login (e.g., Kelley and Bertenthal, 2016b). The findings from the current study confirm this intuition by showing that survey-based measures utilizing knowledge and familiarity, while better than the two-factor model, only improved performance by 2%. Mouse tracking is an especially valuable measure of user behavior because it is capable of capturing the dynamics of perception, categorization, and decision-making (Goodale et al., 1986; Cisek and Kalaska, 2005; Song and Nakayama, 2006; McKinstry et al., 2008; Freeman et al., 2011). As such, the inclusion of this measure not only increases the predictive validity of the model, but offers additional insights into the decision making process associated with threat detection at websites.

Unlike previous mouse-tracking studies, the current study introduced a normalization procedure for extending mouse trajectories to paradigms that do not have fixed and symmetrically positioned alternative choices (Kelley and Bertenthal, 2016a). More specifically, we normalized the distances between start and end locations of the mouse trajectory by converting distance to a unit vector and then rotating the trajectory to a standard location. In addition, we demonstrated that it was possible to extend mouse tracking research to crowd-sourcing websites in order to collect data. Lastly, converging measures of mouse trajectories were used to examine user decision-making, which examined different facets of human behavior. These findings highlight the rich amount of information that can be derived from mouse movement recordings during cognitive tasks.

### Limitations

The present study compared the accuracy of two-factor, survey-based, and real-time measures in predicting risky online behavior during phishing attacks. This study was limited in requiring users to interact with simulated website scenarios. While this format provided experimental control within a semi-naturalistic framework, the format necessarily constrained participant responses. In particular, participants interacted with image-mapped versions of websites that only had limited functionality. Thus, participants could only click on login account links, then either the back button or login button on each website. Furthermore, while most participants reported using Firefox as their primary browser, requiring participants to utilize a Firefox browser may have also affected participant results (Kelley and Bertenthal, 2016b).

Another potential limitation was the difference in penalty times in skipping a legitimate web site and logging into a bad website. The initial difference was created to address issues with participants pressing the simulated back button on the first displayed page. Although that possibility was removed, the penalty time difference was not adjusted. This may have encouraged participants to login at a higher rate, since the penalty was lower.

There are additional confounds regarding the mouse-tracking measures. It was unclear whether some participants used touch pads, keyboard shortcuts, or a mouse while completing the study on Amazon’s Mechanical Turk, which may have created additional variability in results. In addition, while SE was not correlated with a given response (login or back), AUC was. This could mean that AUC is more of a measure of the differences between a login motion and a back motion than the underlying cognitive processes. Longer responses were associated with decisions to back out of versus log in to websites. This could be due to larger distances between the initial login location and the simulated back button over the initial login and the final login button.

Finally, each participant was only presented with a single trial in each condition. This leads to potential limitations in the model analysis due to high individual variances In particular, in predicting responses, there are only six trials to predict, and each trial is essentially a coin flip, it is not too difficult to correctly predict a fair number of responses at random, particularly given that each participants’ intercept is close to the proportion they got correct.

## Conclusion

In order to progress toward understanding more complex and realistic scenarios, cyber security research has begun to utilize behavioral measures, including those that can be examined dynamically. An important step in advancing these measures involves establishing their reliability and validity in predicting and understanding user behavior. The current research demonstrates a semi-naturalistic framework for examining web behaviors experimentally and ethically. Findings validate three widely applicable measures of user behavior derived from mouse recordings, which can be utilized in research and possible user intervention research. Survey data alone are not as strong at predicting risky Internet behavior as models that incorporate real-time measures of user behavior, such as mouse tracking.

## Ethics Statement

This study was carried out in accordance with APA standards on ethical treatment of participants, as well as procedures approved by Indiana University’s Internal Review Board. All subjects gave informed consent in accordance with the Declaration of Helsinki. The protocol was approved by Indiana University’s Internal Review Board.

## Author Contributions

TK contributed to experimental design, data collection, analysis, interpretation, and writing. MA contributed to interpretation of results, writing, and manuscript organization. BB contributed to experimental design, project supervision, and writing.

## Conflict of Interest Statement

The authors declare that the research was conducted in the absence of any commercial or financial relationships that could be construed as a potential conflict of interest.
